# Supplementary material for: Transcriptome Analysis Reveals the Early Development in Subcutaneous Adipose Tissue of Laiwu Piglets
Source: Animals (Basel). 2024 Oct 14;14(20):2955. doi: 10.3390/ani14202955 (PMC11506143; doi:10.3390/ani14202955)
Supplement: Supplementary file 1 [file animals-14-02955-s001.zip › Additional file 1.pdf]

Supplemental Table S1. DEGs in mitochondria function

Gene ID	Gene Symbol	male 21d vs. 1d		female 21d vs. 1d	
		Log2(FC)	Padjust	Log2(FC)	Padjust
ENSSSCG00000018065	ND1	-0.653	6.04×10^{-3}	-1.003	8.83×10^{-5}
ENSSSCG00000018069	ND2	-0.577	2.53×10^{-2}	-1.156	5.29×10^{-6}
ENSSSCG00000018084	ND3	-0.782	2.62×10^{-4}	-1.407	6.35×10^{-11}
ENSSSCG00000018087	ND4	-0.823	1.42×10^{-3}	-1.183	2.45×10^{-8}
ENSSSCG00000018091	ND5	-1.071	4.05×10^{-5}	-1.297	1.09×10^{-9}
ENSSSCG00000018092	ND6	-0.866	2.46×10^{-3}	-1.511	1.48×10^{-11}
ENSSSCG00000016877	NNT	-1.363	2.14×10^{-10}	-1.182	7.32×10^{-5}
ENSSSCG00000025881	PDHB	-1.980	2.91×10^{-8}	-1.859	9.70×10^{-7}
ENSSSCG00000013298	PDHX	-1.119	5.49×10^{-6}	-1.413	1.51×10^{-9}
ENSSSCG00000025486	MDH2	-1.329	1.76×10^{-8}	-1.481	2.05×10^{-5}
ENSSSCG00000015030	DLAT	-1.518	6.22×10^{-9}	-1.964	6.76×10^{-12}
ENSSSCG00000037869	NDUFA5	-0.793	4.56×10^{-4}	-1.183	1.15×10^{-7}
ENSSSCG00000016100	NDUFB3	-0.971	2.14×10^{-4}	-1.115	3.52×10^{-4}
ENSSSCG00000016127	NDUFS1	-1.335	9.90×10^{-13}	-1.768	2.90×10^{-11}
ENSSSCG00000004499	ATP5F1A	-1.217	5.46×10^{-7}	-1.421	1.66×10^{-5}
ENSSSCG00000017544	ATP5MC1	-1.195	2.50×10^{-4}	-1.414	3.35×10^{-4}
ENSSSCG00000015973	ATP5MC3	-0.926	4.28×10^{-4}	-1.209	1.45×10^{-5}
ENSSSCG00000014289	UQCRQ	-1.008	8.01×10^{-6}	-1.216	3.48×10^{-5}
ENSSSCG00000027085	UQCRFS1	-1.072	1.25×10^{-6}	-1.370	1.07×10^{-6}

ID: identifier, FC: fold change.
