# Supplementary material for: Transcriptome Analysis Reveals the Early Development in Subcutaneous Adipose Tissue of Laiwu Piglets
Source: Animals (Basel). 2024 Oct 14;14(20):2955. doi: 10.3390/ani14202955 (PMC11506143; doi:10.3390/ani14202955)
Supplement: Supplementary file 1 [file animals-14-02955-s001.zip › Additional file 2.pdf]

Figure S1

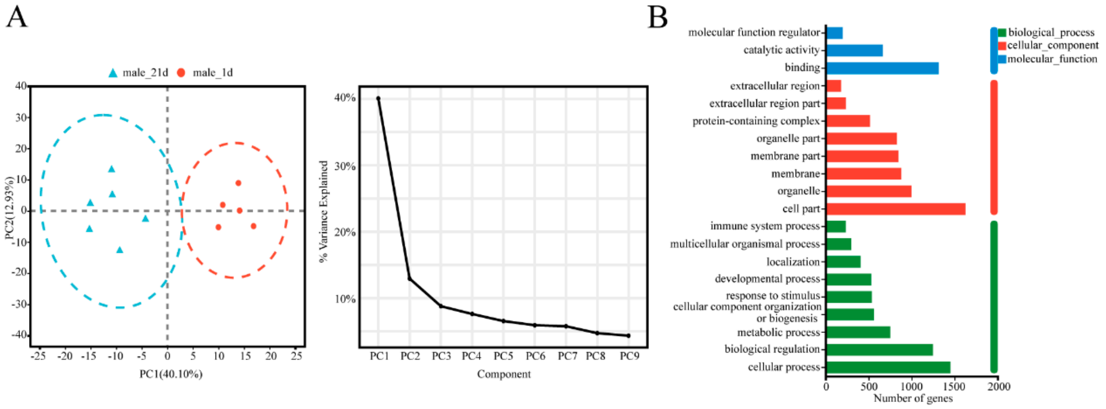

Figure S1. PCA and GO analysis of SAT in male Laiwu piglets. **(A)** Principal component analysis (PCA) of DEGs between 1-d and 21-d SAT. **(B)** GO terms enrichment of DEGs.

Figure S2

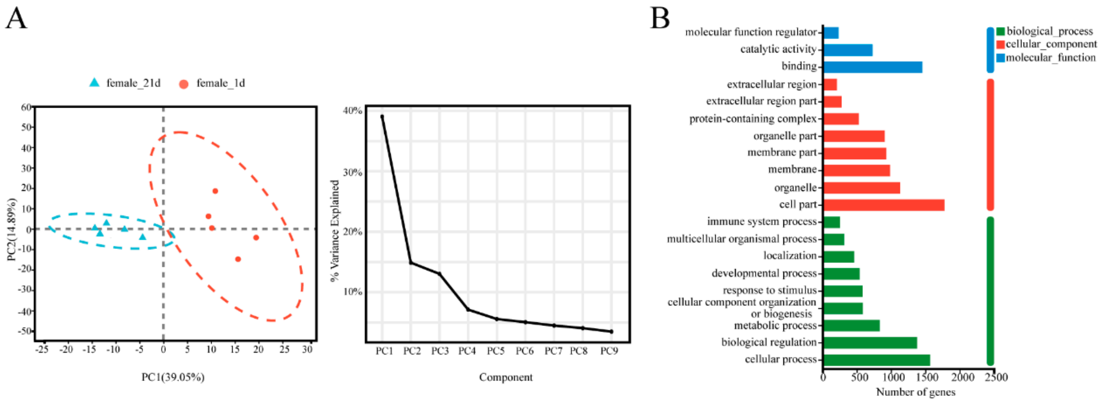

Figure S2. PCA and GO analysis of SAT in female Laiwu piglets. **(A)** PCA of DEGs between 1-d and 21-d SAT. **(B)** GO terms enrichment of DEGs.

Figure S3

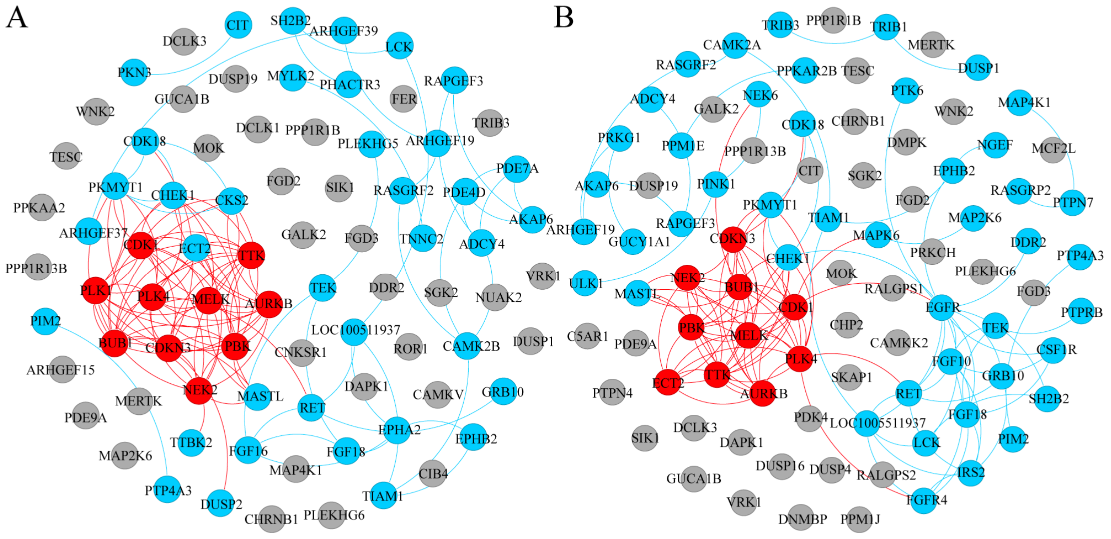

Figure S3. PPI network analysis of SAT in male and female Laiwu piglets. **(A)** PPI diagram of DEGs in male Laiwu piglets SAT in signal transduction mechanisms category of COG classification. **(B)** PPI diagram of DEGs in female Laiwu piglets SAT in signal transduction mechanisms category of COG classification.

Figure S4

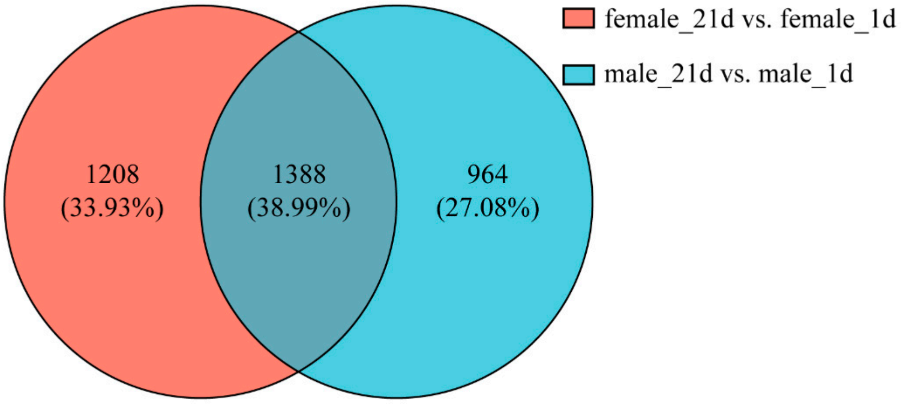

Figure S4. The venn diagram of DEGs between 1-d and 21-d in SAT of male and female Laiwu piglets.

Figure S5

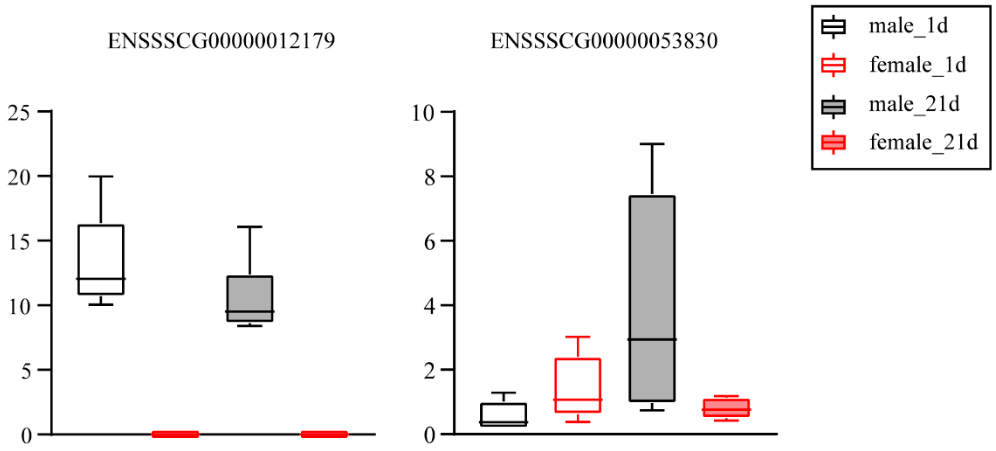

Figure S5. Expression levels of differentially expressed genes between male and female in SAT of 1-d and 21-d old Laiwu piglets. Red box represents female, black box represents male, with the light and dark shades of the same color representing 1-day and 21-day, respectively.
